# Impact of age on hospital outcomes after catheter ablation for ventricular tachycardia

**DOI:** 10.1002/joa3.12998

**Published:** 2024-02-05

**Authors:** Min Choon Tan, Yong Hao Yeo, Qi Xuan Ang, Chrystina Kiwan, Olubadewa Fatunde, Justin Z. Lee, Aneesh Tolat, Dan Sorajja

**Affiliations:** ^1^ Department of Cardiovascular Medicine Mayo Clinic Phoenix Arizona USA; ^2^ Department of Internal Medicine New York Medical College at Saint Michael's Medical Center Newark New Jersey USA; ^3^ Department of Internal Medicine/Pediatrics William Beaumont University Hospital Royal Oak Michigan USA; ^4^ Department of Internal Medicine Sparrow Health System and Michigan State University East Lansing Michigan USA; ^5^ Department of Cardiovascular Medicine Cleveland Clinic Cleveland Ohio USA; ^6^ Department of Cardiovascular Medicine Hartford Healthcare/University of Connecticut Hartford Connecticut USA

**Keywords:** adult, catheter ablation, elderly, hospital outcome, ventricular tachycardia

## Abstract

**Background:**

The real‐world data on the safety profile of ventricular tachycardia (VT) ablation among elderly patients is not well‐established. This study aimed to evaluate the procedural outcomes among those aged 18–64 years versus those aged ≥65 years who underwent catheter ablation of VT.

**Method:**

Using the Nationwide Readmissions Database, our study included patients aged ≥18 years who underwent VT catheter ablation between 2017 and 2020. We divided the patients into non‐elderly (18–64 years old) and elderly age groups (≥65 years old). We then analyzed the in‐hospital procedural outcome and 30‐day readmission between these two groups.

**Results:**

Our study included 2075 (49.1%) non‐elderly patients and 2153 (50.9%) elderly patients who underwent VT ablation. Post‐procedurally, elderly patients had significantly higher rates of prolonged index hospitalization (≥7 days; 35.5% vs. 29.3%, *p* < .01), non‐home discharge (13.4% vs. 6.0%, *p* < .01), 30‐day readmission (17.0% vs. 11.4%, *p* < .01), and early mortality (5.5% vs. 2.4%, *p* < .01). There was no significant difference in the procedural complications between two groups, namely vascular complications, hemopericardium/cardiac tamponade, cerebrovascular accident (CVA), major bleeding requiring blood transfusion, and systemic embolization. Through multivariable analysis, the elderly group was associated with higher odds of early mortality (OR: 7.50; CI 1.86–30.31, *p* = .01), non‐home discharge (OR: 2.41; CI: 1.93–3.00, *p* < .01) and 30‐day readmission (OR: 1.58; CI 1.32–1.89, *p* < .01).

**Conclusion:**

Elderly patients have worse in‐hospital outcome, early mortality, non‐home discharge, and 30‐day readmission following catheter ablation for VT. There was no significant difference between elderly and non‐elderly groups in the procedural complications.

## Graphical abstract

Our study suggests that elderly patients have poorer in‐hospital outcome, early mortality, non‐home discharge, and 30‐day readmissions following catheter ablation for VT. However, elderly patients do not have a higher risk of procedural complications.

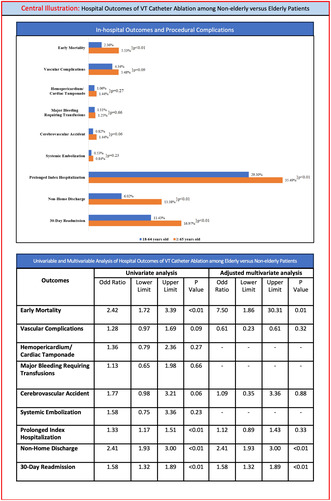

## 1 INTRODUCTION

Catheter ablation for ventricular tachycardia (VT) is increasingly used because of its efficacy in reducing the recurrence of VT and improving long‐term survival as compared to conservative antiarrhythmic therapy.^1^, ^2^ Studies have revealed similar improvement in VT recurrence rate after VT ablation in both elderly and young patient groups.^3^, ^4^ Despite the efficacy, complications following catheter ablation for VT range from 7.1% in non‐ischemic cardiomyopathy to 11.2% in ischemic cardiomyopathy.^5^, ^6^ While there is literature assessing the procedural outcome among elderly patients, these studies are limited to a small scale and thus limit the applicability of findings in these high‐risk patients.^4^, ^7^, ^8^

Therefore, we conducted a nation‐wide retrospective study to determine the impact of age on the in‐hospital outcomes of VT ablation and assess the safety profile among elderly patients.

## 2 METHODS

### 2.1 Data source

The data were obtained from the National Readmission Database (NRD) derived from the Healthcare Cost and Utilization Project (HCUP) State Inpatient Databases. HCUP is sponsored by the Agency for Healthcare Research and Quality. The National Readmission Database is one of the nation's largest publicly available all‐payer inpatient care databases. It is an annual database that includes approximately 17 million discharges yearly from 2017 to 2020. Using verified patient linkage numbers, it can reliably track patient admissions to any hospital in the same state over the course of a year. Based on the International Classification of Diseases, Tenth Revision, Clinical Modification (ICD‐10‐CM) codes, the patient's diagnoses and procedures during each admission were recorded. We queried this database using ICD‐10‐CM codes to identify the patient demographic characteristics, the healthcare facility variables, and the in‐hospital outcomes of each admission. Because NRD is publicly available and de‐identified, our study did not require either institutional review board review or informed consent.

### 2.2 Study population

Using ICD‐10‐CM, we searched for all the patients 18 years of age or above who had a primary diagnosis of VT (I47.2) and underwent catheter ablation for VT (025K3ZZ, 025M3ZZ, 025L3ZZ, 02583ZZ) during the hospitalizations from January 2017 to November 2020. We excluded patients who underwent new pacemaker implantation, open surgical ablation, as well as those having other types of arrhythmias including supraventricular tachycardia, ventricular premature complexes, pre‐excitation syndromes, atrial flutter and atrial fibrillation, in order to ensure a homogenous study population (Table S1). We further divided the patients into non‐elderly (18–64 years old) and elderly age groups (≥65 years old). Patients with missing data for in‐hospital mortality and length of stay were also excluded. As the NRD is constructed using a calendar year of discharge data that does not track the patients over the years, index admissions from December were excluded given that the 30‐day follow‐up after discharge would not be available.

### 2.3 Study endpoints

The primary endpoint of our study was the hospital outcome of patients who underwent catheter ablation for VT. The hospital outcomes included procedural complications (cerebrovascular accident [CVA], major bleeding requiring transfusion, vascular complications, hemopericardium or cardiac tamponade, and systemic embolization), length of hospital stay, hospitalization cost, discharge disposition, 30‐day readmission, and early mortality (combination of the mortality during index admission and readmission within 30 days of procedure). The number of days from the discharge of index hospitalization to the readmission was used to define the time of readmission. If there were multiple readmissions within 30 days after discharge from index hospitalization, only the first readmission was included for analysis. Same‐day transfers within the same hospital or between hospitals are not considered readmissions.

### 2.4 Definition of clinical variables

Patient‐level and hospital‐level variables including age, sex, hospital characteristics (bed size and teaching status), and patient characteristics (median household income based on zip code, primary payer, type of index admission, and discharge disposition) were derived from NRD variables. Patient comorbidity diagnoses were identified by ICD‐10‐CM codes (Table S2). The cost of hospitalization is calculated by adjusting the charge of each hospitalization to the cost‐to‐charge ratio provided by HCUP. We defined cumulative cost of hospitalization as the sum of the cost of index hospitalization and the first 30‐day readmission after VT ablation if readmission occurs.

### 2.5 Statistical analysis

Continuous data were summarized as mean, standard deviation, median, interquartile range (Q1, Q3), and range; differences between groups were tested using Wilcoxon Rank Sum tests. Categorical data were summarized as counts and percentages; differences between groups were tested using Pearson's chi‐squared test. All tests were 2‐sided with p values **≤**0.05 indicating statistical significance. Statistical analyses were conducted by using Stata version 12.1 (Stata Corporation, College Station, Texas). We first conducted propensity‐score matching with 1:1 matching and a caliper of 0.2 on the study's patient population. Subsequent analyses were performed based on this matched patient data. All variables in Table 1 were analyzed based on the in‐hospital outcomes using weighted univariable analyses. Variables with a *p*‐value of <.1 from the univariable analysis were then included in weighted multivariable logistic regression by taking into account the cluster, strata and weighting design of NRD. ROC curve was plotted to measure the area under the ROC curve for the assessment of the accuracy of our multivariable model. Cochrane Armitage test was used to assess the trends of categorical variables and simple linear regression was used to assess the trends of continuous variables.

**TABLE 1:** Baseline patient and hospital characteristics for patients who underwent catheter ablation for ventricular tachycardia.

	18–64 years old		≥65 years old		*p*‐value
	*n*	%	*n*	%	
No. of admissions	2075	49.08	2153	50.92	
**Baseline characteristics**					
Age, mean (SD), y	52.16 (10.68)		73.09 (5.68)		
Female sex	531	25.59	303	14.07	<.01
Alcohol abuse	85	4.10	36	1.67	<.01
Anemia	49	2.36	64	2.97	.22
Chronic kidney disease	334	16.10	748	34.74	<.01
Chronic liver disease	81	3.90	93	4.32	.50
Chronic pulmonary disease	335	16.14	513	23.83	<.01
Coagulation disorder	108	5.20	185	8.59	<.01
Congestive heart failure	1416	68.24	1818	84.44	<.01
Coronary artery disease	1041	50.17	1736	80.63	<.01
Diabetes mellitus	518	24.96	754	35.02	<.01
Hyperlipidemia	995	47.95	1488	69.11	<.01
Hypertension	1308	63.04	1796	83.42	<.01
Malignancy	20	0.96	71	3.30	<.01
Non‐ischemic cardiomyopathy	492	23.71	238	11.05	<.01
Obesity	501	24.14	320	14.86	<.01
Obstructive sleep apnea	365	17.59	365	16.95	.58
Peripheral arterial disease	1029	49.59	1364	63.35	<.01
Prior coronary artery bypass graft	225	10.84	649	30.14	<.01
Prior implantable cardioverter defibrillator placement	978	47.13	1258	58.43	<.01
Prior myocardial infarction	550	26.51	855	39.71	<.01
Prior pacemaker placement	47	2.27	71	3.30	.04
Prior percutaneous coronary intervention	397	19.13	617	28.66	<.01
Prior stroke/transient ischemic attack	124	5.98	208	9.66	<.01
Pulmonary hypertension	110	5.30	138	6.41	.13
Smoking	913	44.00	1023	47.52	.02
Substance use disorder	106	5.11	25	1.16	<.01
Valvular heart disease	262	12.63	371	17.23	<.01
Elixhauser comorbidity score					
<4	692	33.35	312	14.49	<.01
≥4	1383	66.65	1841	85.51	
Charlson comorbidity index (%)					
0	372	17.93	112	5.20	<.01
1	344	16.58	212	9.85	
≥2	1359	65.49	1829	84.95	
Median household income					
First quartile	462	22.27	448	20.81	.70
Second quartile	529	25.49	540	25.08	
Third quartile	566	27.28	599	27.82	
Fourth quartile	495	23.86	529	24.57	
Primary payer					
Medicare	489	23.57	1869	86.81	<.01
Medicaid	300	14.46	16	0.74	
Private including health maintenance organization	1135	54.70	183	8.50	
Self‐pay/no charge/others	145	6.99	83	3.86	
Non‐elective index admission	1599	77.06	1661	77.15	.94
**Hospital variables**					
Hospital size					
Small	73	3.52	87	4.04	.09
Medium	339	16.34	400	18.58	
Large	1663	80.14	1666	77.38	
Hospital procedural volume					
1st quartile	63	3.04	66	3.07	.10
2nd quartile	137	6.60	182	8.45	
3rd quartile	367	17.69	398	18.49	
4th quartile	1508	72.67	1507	70.00	
Length of index hospital stay, mean (SE), d	6.19 (7.71)		6.87 (8.06)		<.01
Prolonged index hospital stay (Length of stay, d ≥ 7)	608	29.30	764	35.49	<.01
Disposition					
Home	1950	93.98	1865	86.62	<.01
Facility	76	3.66	195	9.06	
Against medical advice/unknown	49	2.36	93	4.32	
Cumulative cost of hospitalization, median (IQR)	$40 230 ($28,270–$60 903)		$37 612 ($26 374–$56 066)		<.01

## 3 RESULTS

### 3.1 Study population

After propensity‐score matching, our study included 4228 patients who underwent catheter ablation of VT between January and November (2017–2020). Of these, 2075 (49.1%) were non‐elderly patients (52.2 ± 10.7 years of age, 25.6% female) and 2153 (50.9%) were elderly patients (73.1 ± 5.7 years of age, 14.1% female). Table 1 shows the baseline characteristics and hospital characteristics of both patient groups (after adjustment).

### 3.2 Procedural outcomes

**FIGURE 1:**
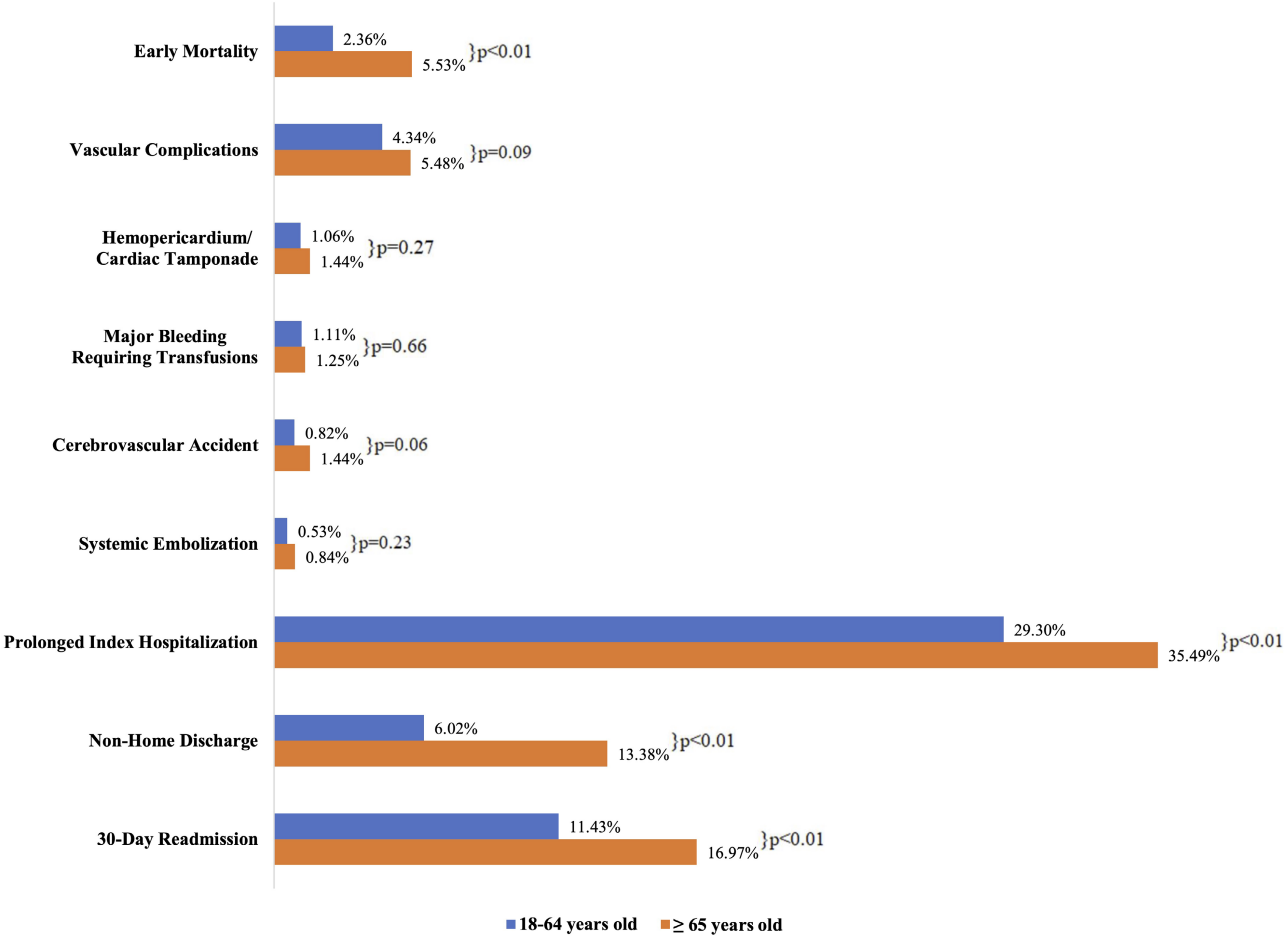
In‐hospital outcomes among patients in different age groups who underwent catheter ablation for ventricular tachycardia.

The procedural outcomes between non‐elderly and elderly groups were depicted in Figure 1. Between these two groups, elderly group had a significantly higher rate of prolonged index hospitalization (≥7 days; 35.5% vs. 29.3%, *p* < .01), non‐home discharge (13.4% vs. 6.0%, *p* < .01), 30‐day readmission (17.0% vs. 11.4%, *p* < .01), and early mortality (5.5% vs. 2.4%, *p* < .01). The median cumulative cost of hospitalization was also significantly higher in the elder groups ($40 230 vs. $37 612, *p* ≤ .01). There was no significant difference between elderly and non‐elderly groups in procedural complications, namely vascular complications (5.5% vs. 4.3%, *p* = .09), hemopericardium or cardiac tamponade (1.4% vs. 1.1%, *p* = .27), CVA (1.4% vs. 0.8%, *p* = .06), major bleeding requiring blood transfusion (1.3% vs. 1.1%, *p* = .66), and systemic embolization (0.8% vs. 0.5%, *p* = .23; Table 2).

**TABLE 2:** Univariate and multivariate analysis of outcomes of 18–64 years versus ≥65 years in catheter ablation for ventricular tachycardia.

Outcomes	18–64 years old, *n* (%)	≥65 years old, *n* (%)	Univariate analysis				Adjusted multivariate analysis			
			Odd ratio	Lower limit	Upper limit	*p* value	Odd ratio	Lower limit	Upper limit	*p* value
Early mortality	49 (2.4%)	119 (5.5%)	2.42	1.72	3.39	<.01	7.50	1.86	30.31	.01
Vascular complications	90 (4.3%)	118 (5.5%)	1.28	0.97	1.69	.09	0.61	0.23	0.61	.32
Hemopericardium/ cardiac tamponade	22 (1.1%)	31 (1.4%)	1.36	0.79	2.36	.27	–	–	–	–
Major bleeding requiring transfusions	23 (1.1%)	27 (1.3%)	1.13	0.65	1.98	.66	–	–	–	–
Cerebrovascular accident	17 (0.8%)	31 (1.4%)	1.77	0.98	3.21	.06	1.09	0.35	3.36	.88
Systemic embolization	11 (0.5%)	18 (0.8%)	1.58	0.75	3.36	.23	–	–	–	–
Prolonged index hospitalization	608 (29.3%)	764 (35.5%)	1.33	1.17	1.51	<.01	1.12	0.89	1.43	.33
Non‐home discharge	125 (6.0%)	288 (13.4%)	2.41	1.93	3.00	<.01	2.41	1.93	3.00	<.01
30‐Day readmission	233 (11.4%)	350 (17.0%)	1.58	1.32	1.89	<.01	1.58	1.32	1.89	<.01

### 3.3 Trend in 30‐day readmission after catheter ablation for VT

**FIGURE 2:**
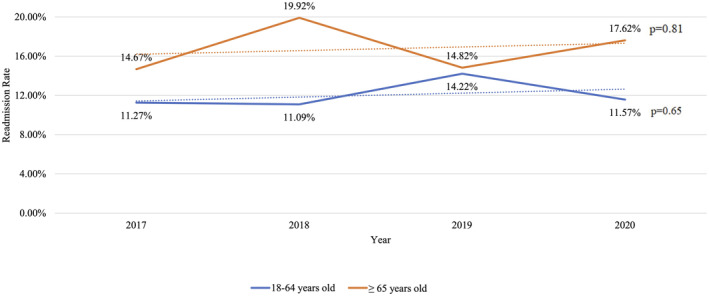
Trends in 30‐day readmissions rate after catheter ablation for ventricular tachycardia.

Over the study period, a non‐significant increasing trend was seen in 30‐day readmission rate after VT ablation in both non‐elderly (11.3% in 2017 to 11.6% in 2020, *p* = .65) and elderly groups (14.7% in 2017 to 17.6% in 2020, *p* = .81; Figure 2).

### 3.4 Predictors of hospital outcome and procedural complications

Through multivariate analysis, the elderly group was associated with significantly higher odds of early mortality (Odd Ratios [OR]: 7.50; 95% Confidence Interval [CI] 1.86–30.31, *p* = .01), non‐home discharge (OR: 2.41; CI: 1.93–3.00, *p* < .01), and 30‐day readmissions (OR: 1.58; CI: 1.32–1.89, *p* < .01; Table 2). Figure S1 demonstrates the early mortality among elderly patient group. Predictors of early mortality in the elderly patient group are depicted in Table S3.

## 4 DISCUSSION

This is the largest all‐payer data in the United States from a nationwide readmission database on in‐hospital outcomes of patients of different age groups who underwent catheter ablation for VT. Our analysis showed that (1) elderly patients were associated with a higher risk of early mortality, non‐home discharge, and 30‐day readmissions after catheter ablation for VT; and (2) there was no significant difference between elderly and non‐elderly groups in the procedural complications.

In‐hospital and procedural outcomes following a procedure are objective indicators of the procedure's safety. Thus, it is crucial to evaluate the patients' outcome post‐procedurally, to improve procedural efficacy and safety. The higher early mortality rate in the elderly patients seen in our study is in line with existing studies.^3^, ^9^ PAINESD scoring tool is used to stratify the risk of acute hemodynamical instability during the VT ablation. A score ≥15 has been suggested as a prognostic indicator of early mortality post‐procedurally.^10^, ^11^, ^12^ While older age is considered a poor prognostic factor, chronic obstructive pulmonary disease, ischemic cardiomyopathy, heart failure, VT storm, and diabetes mellitus are also within the PAINESD scoring system.^11^ In our cohort, patients in the elderly group have higher rates of prevalence in those comorbidities. With the higher burden of comorbidities, the elderly groups represent a sicker group of patients. This predisposes them to a higher risk of early mortality following VT ablation. Thus, careful pre‐procedural planning and post‐procedural management are necessary to optimize the procedural outcome of these elderly patients.

The higher early mortality of elderly patients post‐procedurally could also be because of the higher prevalence of ischemic heart disease in this patient group. More than 80% of elderly patients who underwent VT ablation had a history of coronary artery disease, which was 1.6 times higher than the younger patients. In addition to aging‐related myocardial fibrosis, the scar tissues formed post‐infarction are additional arrhythmogenic substrates for VT.^13^, ^14^ These predispose to development of sustained and recurrent VT.^15^ While the procedural complication profiles were similar between these two groups, the reasons for elderly patients having a higher early mortality rate could be because of more substrate and its related VT and more rapid deterioration from the disease itself rather than from the catheter ablation procedure. This assumption is further supported by a study revealing that recurrent VT was the most common cause of early mortality after catheter ablation for VT.^16^ A higher prevalence of heart failure among the elderly cohort might also contribute to the higher early mortality observed, given that a lower ejection fraction was associated with post‐VT ablation early mortality and decompensated heart failure was the second most common cause of death.^16^

Clinicians tend to avoid invasive procedures in the elderly because of the concern of a higher risk of procedural complications owing to cardiovascular structural changes seen in the aging process.^17^, ^18^, ^19^ However, our study revealed no difference in short‐term procedural complications between elderly and non‐elderly patient groups. This finding is consistent with existing literature.^3^, ^8^, ^17^, ^20^ Similar findings were also found in other cardiovascular procedures such as catheter ablation for atrial fibrillation and implantation of implantable cardioverter‐defibrillator.^21^, ^22^ These suggest that advanced age should not be a factor precluding VT ablation in elderly patients. Despite a similar procedural safety profile in the short‐term, consideration should still be given for close peri‐operative monitoring because of the higher rate of 30 readmissions and early mortality, which may be addressable.

The major advantage of this study is the large sample size included. We believe that our study consisting of 4228 patients from hospitals of different sizes and levels in the United States can provide a real‐world data that is applicable not only to tertiary centers but also those centers with lower procedural volume. The procedural complication data not only helps with pre‐procedure risk assessments but also serves as an important information for post‐procedural monitoring.

## 5 LIMITATIONS

Despite routine quality‐control measures by HCUP to ensure the data validity and reliability, there are still some limitations in our study. Firstly, as with most of the large administrative database studies, the main limitation includes miscoding in primary diagnoses and under‐reporting of secondary diagnoses. Secondly, the out‐of‐hospital deaths that occurred prior to readmission are not recorded, which limits our early mortality to in‐hospital mortality. Thirdly, specific patient variables such as left ventricular ejection fraction, medications, and procedural characteristics such as type of anesthesia, procedural duration, VT inducibility, VT mappability, ablative strategy, and location are not available. The types of VT are unable to be differentiated. These limit our attempts to explore the impact of VT substrate and catheter ablation on procedural outcomes. Fourthly, the confounding effect by the significant differences in demographics between the non‐elderly and elderly cohort might affect the outcomes observed, despite our efforts of performing multivariable analysis and propensity score matching to reduce this bias. The last limitation of our study is not being able to track the patients who are admitted in one state and readmitted in another state.

## 6 CONCLUSION

Our study suggests that despite poorer in‐hospital outcomes, elderly patients do not have a higher risk of procedural complications. These emphasize the similar safety profile of VT ablation in both elderly and non‐elderly patients but also highlight the importance of the need for careful post‐ablation management in these elderly patients.

## DISCLOSURES

All authors have no relationships relevant to the contents of this paper to disclose.

## FUNDING INFORMATION

This research did not receive any specific grant from funding agencies in the public, commercial, or not‐for‐profit sectors.

## CONFLICT OF INTEREST STATEMENT

Authors declare no conflict of interests for this article.

## Supporting information


Data S1.
