# Supplementary material for: Impact of age on hospital outcomes after catheter ablation for ventricular tachycardia
Source: J Arrhythm. 2024 Feb 5;40(2):317–24. doi: 10.1002/joa3.12998 (PMC10995587; doi:10.1002/joa3.12998)
Supplement: Supplementary file 1 — Data S1. [file JOA3-40-317-s001.docx]

Supplementary Figure and Tables

Supplementary Figure 1. Early Mortality among Elderly Patients Who Received Catheter Ablation of VT.


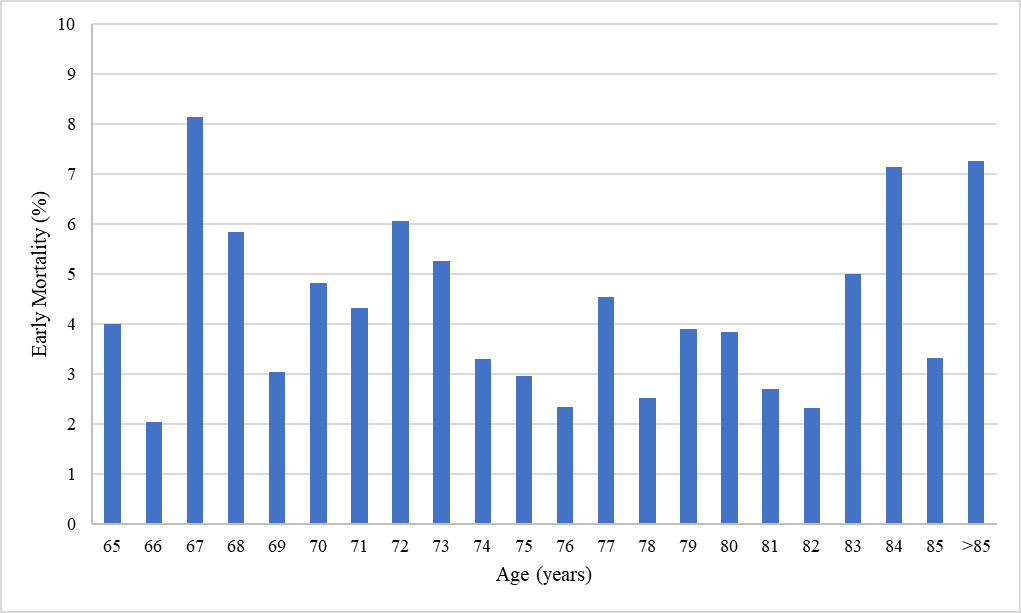


Supplementary Table 1. International Classification of Diseases, Tenth Revision, Clinical Modification (ICD-10-CM) codes of Inclusion and Exclusion Criteria.

| **Inclusion criteria** | **ICD-10-CM/PCS** |
| --- | --- |
| Ventricular tachycardia | I47.2 |
| Catheter Ablation for VT | 025K3ZZ, 025M3ZZ, 025L3ZZ, 02583ZZ |
| **Exclusion criteria** | **ICD-10-CM/PCS** |
| Atrial Fibrillation | I48.0, I48.1, I48.2, I48.91 |
| Atrial flutter | I48.3, I48.4, I48.92 |
| Supraventricular Tachycardia | I47.1 |
| Ventricular premature complexes | I49.3 |
| Pre-excitation syndrome | I45.6 |
| Pacemaker implantation | 02H40JZ, 02H40NZ, 02H43JZ, 02H43NZ, 02H44JZ, 02H44NZ, 02H60JZ, 02H60NZ, 02H63JZ, 02H63NZ, 02H64JZ, 02H64NZ, 02H70JZ, 02H70NZ, 02H73JZ, 02H73NZ, 02H74JZ, 02H74NZ, 02HK0JZ, 02HK0NZ, 02HK3JZ, 02HK3NZ, 02HK4JZ, 02HK4NZ, 02HL0JZ, 02HL0NZ, 02HL3JZ, 02HL3NZ, 02HL4JZ, 02HL4NZ, 02HN0JZ, 02HN3JZ, 02HN4JZ, 0JH604Z, 0JH605Z, 0JH606Z, 0JH607Z, 0JH634Z, 0JH635Z, 0JH636Z, 0JH637Z, 0JH804Z, 0JH805Z, 0JH806Z, 0JH807Z, 0JH834Z, 0JH835Z, 0JH835Z, 0JH836Z, 0JH837Z |
| Open surgical ablation | 02580ZZ, 025K0ZZ, 025L0ZZ, 025M0ZZ |

Supplementary Table 2. International Classification of Diseases, Tenth Revision, Clinical Modification (ICD-10-CM) codes of Variables Included.

| **Comorbidities** | **ICD-10-CM** |
| --- | --- |
| Alcohol Abuse | F10, E52, G62.1, I426, K29.2, K70.0, K70.3, K70.9, T51, Z50.2, Z71.4, Z72.1 |
| Anemia | D50.0, D50.8, D50.9, D51, D52, D53 |
| Chronic Kidney Disease | I12.0, I13.1, N18, N19, N25.0, Z49.0, Z49.1, Z49.2, Z94.0, Z99.2 |
| Chronic Liver Disease | B18, I85, I86.4, I98.2, K70, K71.1, K713, K714, K71.5, K71.7, K72, K73, K74, K76.0, K76.2, K76.3, K76.4, K76.5, K76.6, K76.7, K76.8, K76.9, Z94.4 |
| Chronic Pulmonary Disease | I27.8, I27.9, J40, J41, J42, J43, J44, J45, J46, J47, J60, J61, J62, J63, J64, J65, J66, J67, J68.4, J70.1, J70.3 |
| Coagulation Disorder | D65, D66, D67, D68, D691, D693, D694, D695, D696 |
| Coronary Artery Disease | I25.1, I25.7, I25.8, I25.9, I25.5 |
| Non-ischemic Cardiomyopathy | A36.81, B33.24, E85.4, I42.0, I42.1, I42.2, I42.3, I42.4, I42.5, I42.6, I42.7, I42.8, I42.9, I43, O90.3, I09.9, I11.0, I13.0, I13.2, I42.0, I42.5, I42.6, I42.7, I42.8, I42.9, I43, I50, P29.0 + excluding I20 to I25 |
| Diabetes Mellitus | E10.0, E10.1, E10.9, E11.0, E11.1, E11.9, E12.0, E12.1, E12.9, E13.0, E13.1, E13.9, E14.0, E14.1, E14.9,  E10.2, E10.3, E10.4, E10.5, E10.6, E10.7, E10.8, E11.2, E11.3, E11.4, E11.5, E11.6, E11.7, E11.8, E12.2, E12.3, E12.4, E12.5, E12.6, E12.7, E12.8, E13.2, E13.3, E13.4, E13.5, E13.6, E13.7, E13.8, E14.2, E14.3, E14.4, E14.5, E14.6, E14.7, E14.8 |
| Congestive Heart Failure | I09.9, I11.0, I13.0, I13.2, I25.5, I42.0, I42.5, I42.6, I42.7, I42.8, I42.9, I43, I50, P29.0 |
| Hyperlipidemia | E78.0, E78.4, E78.1, E78.2, E78.5 |
| Hypertension | I10, I11, I12, I13, I15 |
| Malignancy | C00-C26, C30-C34, C37-C41, C43, C45-C58, C60-C76, C81-C85, C88, C90-C97, C77-C80 |
| Obesity | E66 |
| Obstructive Sleep Apnea | G47.30, G47.31, G47.32, G47.33, G47.34, G47.35, G47.36 G47.37, G47.39 |
| Peripheral Arterial Disease | I70, I71, I73.1, I73.8, I73.9, I77.1, I79.0, I79.2, K55.1, K55.8, K55.9, Z95.8, Z95.9 |
| Prior Coronary Artery Bypass Graft | Z95.1 |
| Prior Implantable Cardioverter Defibrillator Placement | Z95.810 |
| Prior Myocardial Infarction | I25.2 |
| Prior Pacemaker Placement | Z95.0 |
| Prior Percutaneous Coronary Intervention | Z98.61, Z95.5 |
| Prior Stroke/ Transient Ischemic Attack | Z86.73, I69.3 |
| Pulmonary Hypertension | I26, I27, I28.0, I28.8, I28.9 |
| Smoking | F17.200, F17.201, F17.203, F17.208, F17.209, F17.210, F17.211, F17.213, F17.218, F17.219, F17.220, F17.290, F17.299, Z87.891 |
| Substance Use Disorder | F11, F12, F13, F14, F15, F16, F18, F19, Z71.5, Z72.2 |
| Valvular Heart Disease | A52.0, I05, I06, I07, I08, I091, I09.8, I34, I35, I36, I37, I38, I39, Q23.0, Q23.1, Q23.2, Q23.3, Z95.2, Z95.3, Z95.4 |

Supplementary Table 3. Independent Predictors of Early Mortality for Patients ≥65 Years Who Underwent Catheter Ablation of VT.

| **Predictors of Early Mortality for Patients ≥65 Years Who Underwent Catheter Ablation of VT** | **Univariate analysis** | | | | **Adjusted multivariate analysis** | | | |
| --- | --- | --- | --- | --- | --- | --- | --- | --- |
|  | **Odd Ratio** | **Lower Limit** | **Upper Limit** | **P Value** | **Odd Ratio** | **Lower Limit** | **Upper Limit** | **P Value** |
| Chronic kidney disease | 2.99 | 1.96 | 4.58 | <0.01 | 1.79 | 1.10 | 2.92 | 0.02 |
| Chronic liver disease | 8.68 | 5.09 | 14.81 | <0.01 | 3.46 | 1.85 | 6.49 | <0.001 |
| Coagulopathy | 5.22 | 3.26 | 8.37 | <0.01 | 2.12 | 1.22 | 3.67 | 0.01 |
| Prolonged index hospital stay (Length of stay, d ≥7) | 5.99 | 3.71 | 9.68 | <0.01 | 3.85 | 2.31 | 6.41 | <0.01 |
| Hyperlipidemia | 0.38 | 0.25 | 0.58 | <0.01 | 0.51 | 0.32 | 0.82 | 0.01 |
| Anemia | 3.37 | 1.56 | 7.30 | <0.01 | 1.48 | 0.60 | 3.61 | 0.39 |
| Congestive heart failure | 8.77 | 2.15 | 35.79 | <0.01 | 6.36 | 0.84 | 48.40 | 0.07 |
| Coronary artery disease | 0.53 | 0.34 | 0.82 | <0.01 | 0.86 | 0.36 | 2.05 | 0.73 |
| Female | 0.49 | 0.22 | 1.06 | 0.07 | 0.45 | 0.20 | 1.04 | 0.06 |
| Malignancy | 2.54 | 1.13 | 5.70 | 0.02 | 2.20 | 0.83 | 5.85 | 0.11 |
| Non-ischemic cardiomyopathy | 3.18 | 1.97 | 5.15 | <0.01 | 1.72 | 0.66 | 4.47 | 0.27 |
| Peripheral arterial disease | 0.60 | 0.40 | 0.91 | 0.02 | 0.38 | 0.12 | 1.19 | 0.10 |
| Prior Implantable Cardioverter Defibrillator Placement | 0.63 | 0.41 | 0.95 | 0.03 | 1.68 | 0.54 | 5.28 | 0.37 |
| Prior Myocardial Infarction | 0.43 | 0.26 | 0.70 | <0.01 | 0.61 | 0.35 | 1.07 | 0.08 |
| Pulmonary hypertension | 1.82 | 0.92 | 3.59 | 0.09 | 0.80 | 0.36 | 1.78 | 0.59 |
| Smoking | 0.54 | 0.35 | 0.84 | <0.01 | 0.88 | 0.53 | 1.46 | 0.63 |
| Charlson comorbidity index |  |  |  |  |  |  |  |  |
| 1 | 2.13 | 2.36 | 19.33 | 0.50 | 0.06 | 0.01 | 1.55 | 0.09 |
| 2 | 5.61 | 0.77 | 40.65 | 0.09 | 0.10 | 0.01 | 2.40 | 0.15 |
| Elixhauser comorbidity score ≥4 | 16.36 | 2.27 | 117.81 | <0.01 | 9.47 | 0.78 | 115.80 | 0.08 |
